# Fabrication of Stimuli-Responsive Quince/Mucin Co-Poly (Methacrylate) Hydrogel Matrices for the Controlled Delivery of Acyclovir Sodium: Design, Characterization and Toxicity Evaluation

**DOI:** 10.3390/pharmaceutics15020650

**Published:** 2023-02-15

**Authors:** Aysha Aslam, Muhammad Umer Ashraf, Kashif Barkat, Asif Mahmood, Muhammad Ajaz Hussain, Muhammad Farid-ul-Haq, Manar O. Lashkar, Heba A. Gad

**Affiliations:** 1 Faculty of Pharmacy, The University of Lahore, Lahore 54000, Pakistan; 2 Department of Pharmacy, University of Chakwal, Chakwal 48800, Pakistan; 3 Department of Chemistry, The University of Punjab, Lahore 54000, Pakistan; 4 Department of Chemistry, University of Sargodha, Sargodha 40100, Pakistan; 5 Department of Pharmacy Practice, Faculty of Pharmacy, King Abdulaziz University, Jeddah 21589, Saudi Arabia; 6 Department of Pharmaceutics and Industrial Pharmacy, Faculty of Pharmacy, Ain Shams University, Cairo 11566, Egypt; 7 Department of Pharmaceutical Sciences, Pharmacy Program, Batterjee Medical College, Jeddah 21442, Saudi Arabia

**Keywords:** quince, mucin, controlled release, stimuli-responsive, acyclovir sodium, health care, drug discovery

## Abstract

Free-radical polymerization technique was adopted to fabricate a stimuli-responsive intelligent quince/mucin co-poly (methacrylate) hydrogel for the controlled delivery of acyclovir sodium. The developed hydrogel matrices were appraised using different parameters, such as drug loading (%), swelling kinetics, pH- and electrolyte-responsive swelling, and sol–gel fraction. Drug-excipient compatibility study, scanning electron microscopy, thermal analysis, powder X-ray diffraction (PXRD) analysis, in vitro drug release studies, drug release kinetics and acute oral toxicity studies were conducted. The results of drug loading revealed an acyclovir sodium loading of 63–75% in different formulations. The hydrogel discs exhibited pH-responsive swelling behavior, showing maximum swelling in a phosphate buffer with a pH of 7.4, but negligible swelling was obvious in an acidic buffer with a pH of 1.2. The swelling kinetics of the developed hydrogel discs exhibited second-order kinetics. Moreover, the hydrogel discs responded to the concentration of electrolytes (CaCl_2_ and NaCl). The results of the FTIR confirm the formation of the hydrogel via free-radical polymerization. However, the major peaks of acyclovir remain intact, proving drug-excipient compatibility. The results of the SEM analysis reveal the porous, rough surface of the hydrogel discs with multiple cracks and pores over the surface. The results of the PXRD disclose the amorphous nature of the fabricated hydrogel. The dissolution studies showed a minor amount of acyclovir sodium released in an acidic environment, while an extended release up to 36 h in the phosphate buffer was observed. The drug release followed Hixen–Crowell’s kinetics with Fickian diffusion mechanism. The toxicity studies demonstrated the non-toxic nature of the polymeric carrier system. Therefore, these results signify the quince/mucin co-poly (methacrylate) hydrogel as a smart material with the potential to deliver acyclovir into the intestine for an extended period of time.

## 1. Introduction

When using traditional delivery systems, patients tend to be non-compliant and experience side effects from the frequent administration of large doses [1]. Other shortcomings associated with conventional drug delivery systems include fluctuations in plasma drug level, meager bioavailability and gastrointestinal discomfort [2]. Recent advancements in drug delivery systems have successfully countered the flaws associated with conventional dosage forms, such as multiple dosing and gastrointestinal (GI) disorders, and have also contributed to improving patient compliance. Polymer-based drug carriers can be employed to deliver drugs to their desired site at a predetermined rate. In this context, synthetic, semi-synthetic and natural polymers have been extensively used [3] One promising hydrogel system for controlled drug delivery is developed through the chemical entanglement of polymers that form pockets from which drugs slowly diffuse out. Hydrogels are being extensively employed when designing controlled drug delivery systems because of their biocompatibility, biodegradability and stimuli responsiveness [4,5,6]. Hydrogels have profound applications in wound healing [7], tissue engineering, targeted drug delivery [8], biomedical sciences and in vitro diagnostics [9]. Different hydrogel-based carriers, such as discs, nanoparticles, polymeric films and composite matrices, have been developed based on their desired utilization [10,11,12]. Considering the immense utilization of hydrogel-based carriers, we are keen to develop a pH-responsive graft copolymer of quince seed mucilage with mucin for the controlled delivery of acyclovir sodium using free-radical polymerization.

Quince is a glucuronoxylan with a high portion of glucuronic acid. An NMR study revealed 4-O-methyl-α-D-glucopyranosyluronic and α-D-glucopyranosyluronic residues linked with (1→4)-β-D-xylan to position 2 [13,14].

Gastric mucin, a high-molecular-weight glycoprotein, is responsible for providing the gastric mucus layer and performing protective functions. Mucin undergoes a pH-dependent sol–gel transition from a viscoelastic solution at neutral pH to a soft viscoelastic gel under acidic conditions, with the transition occurring near a pH of 4 [15].

Acyclovir sodium is an antiviral medication used to treat herpes simplex virus (HSV), specifically HSV-1 and HSV-2, as well as varicella-zoster virus. It is a BCS class III drug. Acyclovir sodium has a 10–20% oral bioavailability, which further decreases with increasing doses. The terminal plasma half-life of acyclovir sodium after administration in adults is approximately 2.9 h, with the majority of the drug being excreted in an unchanged form by the kidneys [16,17]. In order to circumvent the issues of low bioavailability and frequent dosing with acyclovir, different approaches, such as incorporation of beta cyclodextrin, entrapment in niosomes, mucoadhesive drug delivery system and polymeric nanoparticles, have been developed, but all strategies have their limitations [18].

Commercially available oral tablets of acyclovir containing 200 mg acyclovir/tablet have to be consumed five times a day to achieve the desired therapeutic concentration in HSV infection. Oral administration of such high doses has severe gastrointestinal effects. On the other hand, IV administration of acyclovir may result in kidney damage [19].

Considering the immense utilization of hydrogel-based carriers, the aim of the present study is to fabricate a pH-responsive graft copolymer of quince seed mucilage with mucin for the controlled delivery of acyclovir sodium using free-radical polymerization. The aim of developing a smart stimuli-responsive hydrogel carrier system is to modulate acyclovir release for extended period of time at a predetermined rate, which will not only improve patient compliance but also helpful in addressing the bioavailability issues and unwanted gastrointestinal issues associated with conventional oral therapy. The impact of different variables, i.e., polymers, monomers and cross-linker on swelling, sol–gel fraction (%), drug loading (%) and in vitro drug release (%) of the developed quince/mucin co-poly (methacrylate) hydrogel were investigated. Characterizations using various analytical techniques, such as FTIR, SEM, PXRD and DSC analyses, were also conducted.

## 2. Materials and Methods

### 2.1. Materials

Quince seeds were purchased from Awaami Laboratories Pvt. Ltd., Lahore, Pakistan. NaCl, KCl, HCl, ammonium persulfate and potassium dihydrogen phosphate were provided by Icon Chemicals, Germany, while *n*-hexane was obtained from Merck, Germany. N, N-Methylene bisacrylamide (MBA) was purchased from Thermo Fisher Scientific, Shanghai, China. Methacrylic acid (MAA) was sourced from Duksan Pure Chemicals, Ansan, Republic of Korea. Acyclovir sodium was donated by Trigon Pharmaceuticals Pvt. Ltd., Lahore, Pakistan. In all experimental work, distilled water was used.

### 2.2. Extraction of Quince Hydrogel

Quince seeds (100 g) were immersed in distilled water (500 mL) for 8 h to isolate the quince hydrogel (QH). To maximize the yield, heating at 50 °C for at least 30 min was performed. The extracted QH was separated with a cotton cloth and washed with n-hexane. After washing, the QH was transferred to Petri plates and dried in an hot air oven at 50 °C for 48 h. The dried QH was powdered using a mortar and pestle, sieved through a 60-mesh sieve, and stored in a well-closed plastic jar for further use. The yield of the QH was estimated at 11 gm/100 gm of dried seeds [14].

### 2.3. Fabrication of Quince/Mucin Co-Poly (Methacrylate) Hydrogel

Free-radical polymerization technique was used to fabricate a quince/mucin co-poly (methacrylate) hydrogel. Polymers, monomers and a cross-linker were incorporated at different ratios. The compositions of different formulations (QHM1–QHM12) are presented in Table 1. Specified amounts of each QH were dissolved in deionized water (5 mL) on a magnetic stirrer (VELP Scientifica) until the QH fragments were properly distributed. Similarly, mucin, APS, MAA and MBA were dissolved separately in deionized water (5 mL) on a hot plate magnetic stirrer. The solutions of the polymers (quince and mucin) were mixed together with continuous stirring (100 rpm), followed by APS incorporation. MBA was added to the solution of MAA with continuous stirring. This solution was transferred to the activated polymeric solution with continuous stirring. The whole mixture was sonicated for 5 min, transferred into glass tubes, and sealed with aluminum foil. The test tubes were kept in a thermostatically controlled water bath (Memmert, Tokyo, Japan) at 55 °C for 2 h and then at 65 °C for 8 h. After a specified period of time, the test tubes were removed from the water bath and placed at room temperature for some time. The prepared hydrogel was removed with the help of a spatula and impregnated with ethanol. The cutting of the prepared hydrogels in the form of discs (5 mm thick) was carried out with the help of a sharp blade. Washing was executed with an ethanol and water mixture (70:30) for 30 min to remove unreacted contents. The drying of the discs was accomplished in a hot air oven at 45 °C until a constant weight was achieved [20,21].

### 2.4. Drug Loading (%) 

A preloading procedure was adopted for loading acyclovir sodium into the hydrogel discs [5,6]. Acyclovir sodium (1000 mg) was dissolved in deionized water and incorporated into the mixture of polymeric solutions with continuous stirring on a magnetic stirrer until the drug was uniformly distributed. This polymeric solution was added to the mixture of MAA and MBA with continuous stirring for 30 min. Afterward, the formulation was transferred into a test tube, which was kept in a water bath at 55 °C for 2 h, followed by heating at 65 °C in the water bath for the next 8 h. The drug loading percentage was assessed by comparing the absorbance of the solution of the drug-loaded discs with the absorbance of the standard acyclovir sodium solution on an UV–visible spectrophotometer (Shimadzu, Tokyo, Japan) at 252 nm.

### 2.5. pH-Responsive Swelling Studies

To ascertain the pH responsiveness of the quince/mucin-co-poly (methacrylate) hydrogel, swelling of the hydrogel discs was investigated in an acidic buffer with a pH of 1.2, and a phosphate buffer with a pH of 6.8 or 7.4 at 37 °C. Pre-weighed discs were immersed in the buffer solution, and, after pre-determined time intervals (1, 2, 3, 4, 6, 8, 10, 12, 14, 16, 18, 20, 24, 28, 32 and 36 h), the discs were removed, allowed to drop off excessive immersion medium from their surface, and reweighed. The swelling capacity g/g was calculated using the following equation:

*Swelling capacity* (*g*/*g*) = (*Wt* − *Wo*)/*Wo* (1)

where *Wt* denotes the weight of the disc after time *t,* and *Wo* represents the initial weight of the dried disc.

### 2.6. pH-Responsive Swelling–Deswelling Studies

To establish the stimuli sensitivity of the hydrogel discs, swelling–deswelling studies were conducted using the gravimetric method. For this purpose, the hydrogel discs were immersed in a phosphate buffer with a pH of 7.4 for 1 h and then allowed to deswell in an acidic buffer with a pH of 1.2 for the same amount of time. The weight of the discs during the swelling and deswelling cycles was measured after 15 min intervals for 1 h. The swelling and deswelling studies were executed in repetitive cycles [14]. 

### 2.7. Electrolyte-Responsive Swelling Studies

Molar solutions with different concentrations of sodium chloride and calcium chloride (0.1, 0.2, 0.3, 0.4, 0.5, 1.0 and 2.0 M) were prepared to assess the equilibrium swelling of the quince/mucin co-poly (methacrylate) hydrogel discs. Pre-weighed discs were immersed in the electrolyte solutions for 24 h, after which they were removed from the respective immersion mediums and reweighed on an analytical balance to assess their equilibrium swelling capacities as g/g [22,23]. 

### 2.8. Swelling Kinetics

The swelling kinetics of the hydrogel discs were determined using the normalized degree of swelling (*Q_t_*) and the normalized equilibrium degree of swelling (*Q_e_*). The normalized degree of swelling (*Q_t_*) is the ratio of the weight of a swollen disc at time “*t*” to the weight of a dry disc, and it is calculated using Equation (2) [24]:

(2) $$Qt=\frac{Wt-Wd}{Wd}=\frac{Wt}{Wd}$$

where *W_t_* is the weight of the swollen disc at time “*t*”, and *W_d_* is the weight of the dry hydrogel disc.

The normalized equilibrium degree of swelling (*Q_e_*) is the ratio of the weight of an optimally swollen disc to the weight of a dry disc. It can be calculated using Equation (3) [25]:

(3) $$Qe=\frac{W\infty -Wd}{Wd}=\frac{We}{Wd}$$

where W∞ is the optimally swollen hydrogel disc’s weight, *W_d_* is the weight of the dry hydrogel disc, and *W_e_* is the weight of the disc at equilibrium swelling. 

The swelling kinetics was assessed using Equation (4):

(4) $$\frac{t}{Q_{t}}=\frac{1}{KQe2}=\frac{t}{Qe}$$

Second-order swelling kinetics is confirmed if the graph between *t/Qt* vs. *t* on the y-axis and the x-axis, respectively, is linear [22]. 

### 2.9. Sol–Gel Fraction 

Estimation of the sol–gel fraction was performed to ascertain the utilization of the formulation ingredients in the formulation of the hydrogels. This was established through immersion of pre-weighed discs from different formulations in distilled water (100 mL) at 37 °C for 48 h. The drying of the swollen hydrogel discs was executed in an oven at 45 °C until a constant weight was achieved and then re-weighed. The sol and gel fractions were estimated using the following equation [26,27]:

(5) $$Sol-fraction\;\left(\%\right)=\frac{\left[Wo-Wi\right]}{Wo}\times 100$$

Gel-fraction (%) = 100 − Sol fraction(6)

where *W_o_* refers to the initial weight of the hydrogel disc, and *W_i_* is the final weight of the hydrogel disc. 

### 2.10. Drug-Excipient Compatibility Study

Fourier-transform infrared (FTIR) spectroscopy was used to evaluate the possible interaction of the drug with the used polymers and monomers. This was accomplished using the KBr pellet method on an IR prestige-21 (Shimadzu, Japan). The pellets of the polymers, the unloaded hydrogel discs, the loaded discs and the drug were prepared in a hydraulic press. The drying of the pellets was performed in an oven at 50 °C for 2 h. The spectra were recorded in the transmittance mode from the wavenumber (4000–500 cm^−1^) [28,29]. 

### 2.11. Scanning Electron Microscopy

The surface morphology of the fabricated loaded and unloaded hydrogel discs was ascertained using a scanning electron microscopy (SEM). The swollen freeze-dried discs were trimmed transversely, and their SEM images were recorded at different magnifications using Vega 3, Tusca [29,30]. 

### 2.12. Thermal Analysis 

Differential scanning calorimetry (DSC) was performed to appraise the thermal stability of the individual polymers, the fabricated hydrogel and acyclovir sodium before and after loading into the hydrogel network. The samples were covered in an aluminum pan and were heated over a temperature range (0–400 °C) at a rate of 20 °C/min using a thermal analyzer (Q600 TA V8.3 Build101 Thermal Analysis System, TA Instruments, New Castle, DE, USA) under nitrogen purging at 20 mL/min. All samples were studied in triplicate [31,32].

### 2.13. Powder X-ray Diffraction (PXRD) Analysis

A PXRD analysis was performed to investigate the crystalline/amorphous nature of the polymers and acyclovir sodium before and after loading into the hydrogel network. The samples were analyzed over a scanning range of 2*θ* (10°–70°) using a powder X-ray diffractometer (Bruker, Kahlsruhl, Germany) at room temperature [30,31].

### 2.14. In Vitro Drug Release Studies

A USP type II dissolution apparatus was used to investigate acyclovir sodium release from the polymeric network. The dissolution studies were carried out in an acidic buffer with a pH of 1.2 and phosphate buffers (pH 6.8 and 7.4) for 36 h. To mimic the conditions of the GIT, dissolution studies were also performed on all formulations (QHM1–QHM12) in an acidic buffer with a pH of 1.2 for 4 h, and then a similar disc was shifted to a phosphate buffer with a pH of 6.8 for 10 h. Afterward, dissolution was executed in a buffer solution with a pH of 7.4 for 22 h. A dissolution medium (900 mL) was filled into the baskets, and the drug-loaded discs were placed in each basket. The temperature of the dissolution medium was thermostatically controlled at 37 ± 0.5 °C before placing the discs into the medium. The paddle speed was adjusted at 50 rpm. The samples (5 mL) were removed at predetermined time intervals and analyzed for acyclovir sodium contents using a UV spectrophotometer (Shimadzu, Japan) at a specified scanning range (λ_max_ 252 nm) [4,33].

### 2.15. Drug Release Kinetics

Drug release kinetics was ascertained by applying different kinetic models, i.e., zero-order, first-order, Higuchi, Hixen–Crowell and Korsmeyer–Peppas equations, using the DDSolver add-in program. The value of *R*^2^ was used to determine the best-fit model, while the mechanism of acyclovir sodium release was confirmed by the value of *n* using the Korsmeyer–Peppas equation. If the value of *n* equals 0.45, Fickian diffusion plays a crucial role in the drug release from the polymeric matrices. When the value of *n* > 0.45 < 0.89, non-Fickian diffusion takes place in which drug release is governed by both the diffusion and swelling of the polymeric matrices. If the value of *n* = 0.89 or <0.89, the super case II transport mechanism is involved in the drug release from the polymeric matrices. In this case, a constant amount of drug is released from the polymer chains for a long period of time, and erosion plays a predominant role in the drug release from the polymeric chains [34,35].

The kinetic equations of the different kinetic models are presented below:

Zero-order: *Qt = K_o_ t* (7)

In the above equation, *t* is the time; *K_ᵒ_* is the zero-order rate constant; and *Q_t_* is the amount of drug released from the hydrogel after time *t*.

First-order: *log Q* = *log Q*_0_ − (*K*_1_*t*/*2.303*) (8)

where “*t*” is the time at which drug release is noted; *K_1_* is the first-order rate constant; *Q_0_* represents the initial amount of drug in the hydrogel disc; and *Q* accounts for the drug that is undissolved or still to be released from the hydrogel [36].

Hixen–Crowell model: *Q*_0_^1/^3 − *Qt*1^/^^3^ = −*K_HC_^t^* (9)

where *K_HC_* is the Hixson–Crowell rate constant; *Qo* is the initial amount of drug in the hydrogel; and *Qt* is the amount of drug released from hydrogel after time *t* [37].

Higuchi model: *Qt* = *K_H_* (*t*)^1*/*2^ (10)

Here, *K_H_* is the Higuchi rate constant, and *Q_t_* denotes the amount of drug released from the matrix tablets after time *t* [38].

Korsmeyer–Peppas: *M_t_/M*∞ = *K_p_t^n^* (11)

where *M^t^/M*∞ denotes the amount of drug released in time *t*; *K_p_* is the power-law constant; and *n* denotes the release exponent [39].

### 2.16. Oral Toxicity Studies

To probe the impact of the carrier system on the physical architecture of vital organs, acute oral toxicity studies were executed on rabbits. The study protocols were established according to the Guidelines for Economic Cooperation and Development (OECD). The approval of study protocols was granted by the Institutional Research Ethics Committee of the Faculty of Pharmacy, University of Lahore, vide notification no. IREC-2021-20. The animals (white Albino rabbits) were acquired from the animal house of the University of Lahore one week prior to the execution of the toxicity studies in order to acclimatize them to the lab environment. Proper diet and water access was provided, and all animals were kept in a controlled environment at 25 °C and were kept for 12 h in light and dark conditions in neat and clean cages. 

The animals were divided into three groups, i.e., groups A, B and C (each with three animals). Group A (control) was left untreated, while group B and group C (treated groups) were fed with ground hydrogel discs. They were kept on fasting conditions overnight prior to the administration of the hydrogel discs. Then, a single dose (2 and 4 g/kg) of the powdered disc along with the food was administered to groups B and C, respectively. All animals were kept under strict monitoring of physical health, body weight, food and water consumption, behavioral changes, skin irritation, diarrhea and constipation for 14 days. 

Blood samples were collected on day 14 with the help of a 22-gauge syringe needle from the jugular artery to perform the hematological and biochemical assay. The blood samples were kept in EDTA-lined test tubes in order to avoid clotting and stored in a refrigerator at 4 °C before analysis. Different hematological parameters, such as platelets, neutrophils, monocytes, lymphocytes, red blood cells (RBCs) and white blood cells (WBCs), were analyzed using a hematology analyzer (Beckman Coulter DxH900, Beckman-Coulter, Brea, CA, USA). Plasma from the blood was removed by centrifugation at 4000 rpm for 30 min and analyzed for different biochemical parameters, including alanine aminotransferase (ALT), aspartate aminotransferase (AST), urea, creatinine, cholesterol and triglycerides, using a biochemical analyzer (Microlab 300, ELITech, Puteaux, France). Afterward, the rabbits were sacrificed, and their vital organs, such as heart, liver, lungs, spleen, kidney, intestine and stomach, were removed and preserved in a 10% formalin solution in labeled plastic containers. Histopathological studies were conducted to appraise the effects of the polymeric carriers on the cellular architecture of vital organs [40,41,42].

## 3. Results and Discussion

### 3.1. Drug Entrapment Efficiency

**Figure 1:**
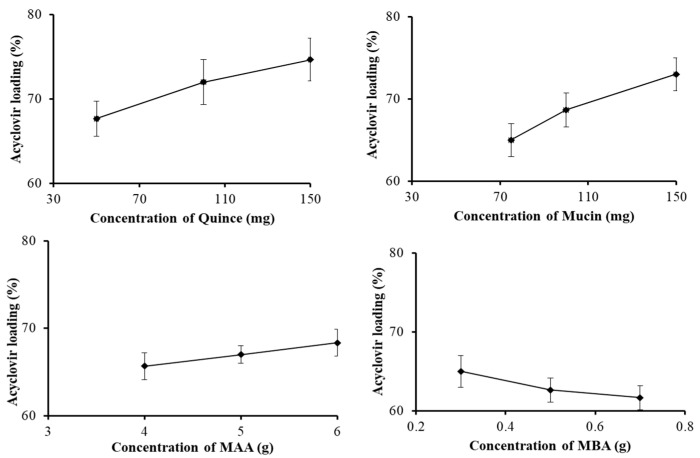
Effect of the concentrations of different formulation components on acyclovir sodium loading (%).

The drug entrapment efficiency of different formulations of the quince/mucin co-poly (methacrylate) hydrogel (QHM1–QHM12) was analyzed; the efficiency ranges from 63 to 75%, as shown in Figure 1.

The results of drug loading (Figure 1) predict that increasing the ratio of quince in the formulations QHM1–QHM3 and the ratio of mucin in the formulations QHM4–QHM6 increases the loading capacity from 67 to 75% and from 65 to 73%, respectively. Similarly, the addition of the monomer (MAA) increases the loading efficiency from 64 to 68% in the formulations QHM7–QHM9. However, by increasing the amount of the cross-linker (MBA), the loading efficiency declines from 65 to 63%. It has been reported that an increase in the ratios of polymers and monomers results in an improved drug-loading [43]. 

The increase in drug loading by increasing the feed contents of quince, mucin and MAA might be attributed to an increase in the gelling contents of the fabricated hydrogel, which results in an increase in the retention of acyclovir sodium within the polymeric network. In case of an increase in the contents of MBA, drug loading decreases, which might be due to an increase in the internal stretch of the polymeric chains. These results comply with previously reported studies [44].

### 3.2. pH-Responsive Swelling Studies

**Figure 2:**
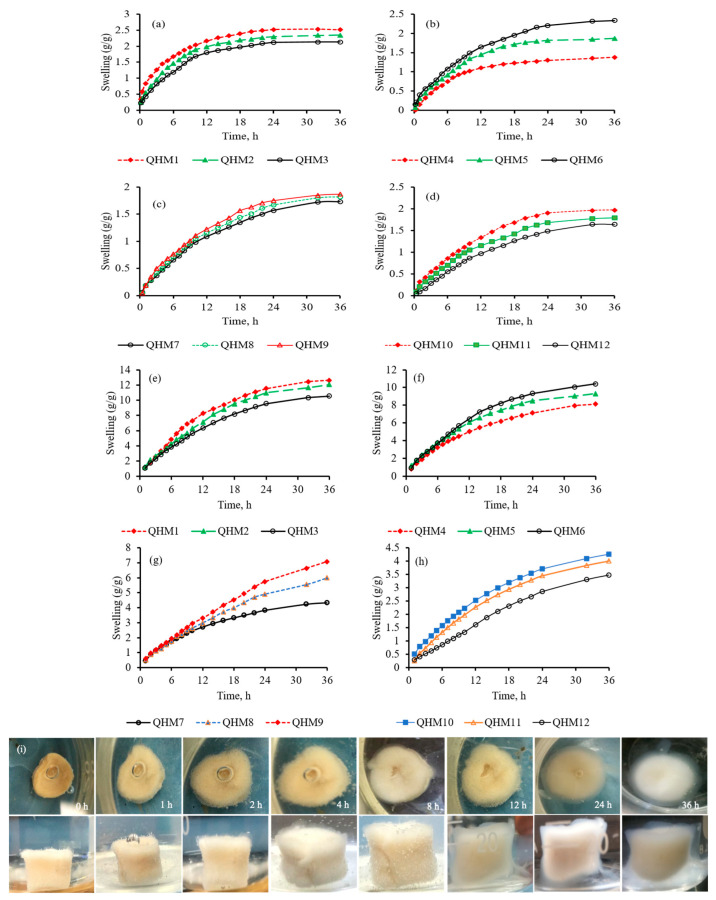
Swelling capacity of hydrogel formulations QHM1–QHM12 in an acidic buffer with a pH of 1.2 (**a**–**d**) and a phosphate buffer with a pH of 7.4 (**e**–**h**). Radial and axial views of the swelling of the hydrogel discs in deionized water at different time intervals (**i**).

Hydrogels possessing a high profound swelling capacity and a rapid rate of swelling are preferred when designing drug delivery systems [45]. In order to estimate the pH sensitivity of the quince/mucin co-poly (methacrylate) hydrogel, the swelling response of different formulations (QHM1–QHM12) were determined in a phosphate buffer with a pH of 7.4 and an acidic buffer with a pH of 1.2. A high swelling profile was witnessed for all formulations in the phosphate buffer with a pH of 7.4, as shown in Figure 2e–h, whereas minor swelling was observed in the acidic buffer with a pH of 1.2, as shown in Figure 2a–d. Moreover, in the phosphate buffer (pH 7.4), it was noticed that a sequential rise in the ratios of quince and mucin in the formulations QHM1–QHM6 resulted in increased swelling from 10 to 13 g/g and from 8 to 10 g/g, respectively. This increase in swelling by increasing quince and mucin is attributed to an increase in hydroxyl and carbonyl groups within the polymeric chains. Due to this increment, repulsive forces within the polymeric chains become more pronounced, allowing more physiological fluid to penetrate into the matrices and resulting in enhanced swelling.

An increase in the amount of methacrylic acid in the formulations QHM7–QHM9 also augmented the swelling response from 5 to 8 g/g in the phosphate buffer with a pH of 7.4. This intensification in swelling by increasing MAA is credited to excessive carboxylic groups present in the MAA. At a pH of 7.4, these carboxylic groups become un-protonated, resulting in an increase in repulsion between the polymeric chains. Consequently, free spaces in the polymeric network increase, and thus more swelling media can penetrate into the hydrogel, which is depicted in the form of increased swelling response [3,46].

Moreover, an incremental rise in MBA in the formulations QHM10–QHM12 resulted in a declination in the swelling response from 4 to 3 g/g, which is credited to the reduced porosity of the polymeric network due to an increase in the cross-linking extent. The reduced porosity halts the diffusion of the swelling medium into the polymeric network, which results in decreased swelling [47].

### 3.3. Swelling Kinetics

**Figure 3:**
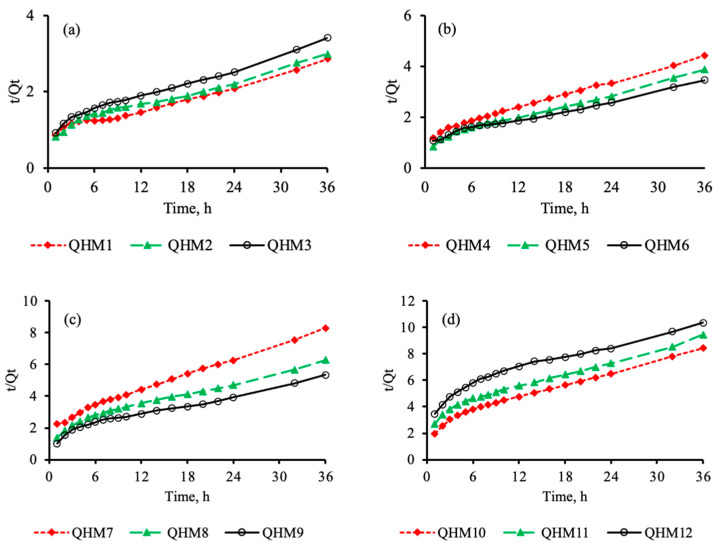
Swelling kinetics of hydrogel formulations QHM1–QHM12 in a phosphate buffer with a pH of 7.4 (**a**–**d**).

To evaluate the swelling kinetics of the fabricated quince/mucin co-poly (methacrylate) hydrogel, the swelling of the hydrogel discs was carried out in a phosphate buffer with a pH of 7.4. A linear relationship between *t/Qt* vs. *t* was noticed, as shown in Figure 3, thereby complying with the second order of swelling kinetics [3,46].

### 3.4. Electrolyte-Responsive Swelling Studies

**Figure 4:**
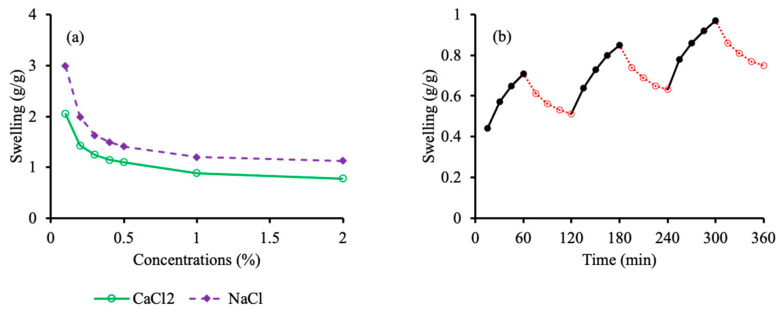
Equilibrium swelling of hydrogel discs in different molar concentrations of NaCl and CaCl_2_ solutions (**a**). Stimuli-responsive swelling/de-swelling of the hydrogel discs in a phosphate buffer with a pH of 7.4 and an acidic buffer with a pH of 1.2 (**b**).

The swelling behavior of the fabricated hydrogel discs was ascertained in aqueous salt solutions of NaCl and CaCl_2_. Solutions with different molar concentrations (0.1, 0.2, 0.3, 0.4, 0.5, 1.0 and 2.0 M) were prepared. The equilibrium swelling of the hydrogel discs rapidly declined as the molar concentration of the salt solutions was increased from 0.1 to 0.4 M. Afterward, a less steep response was noticed from 1.0 to 2.0 M solutions. This decrease in swelling with increasing concentration of electrolytes is attributed to the charge screening effect of the counter ions and a decrease in the osmotic pressure difference between the hydrogel and the salt solution [48]. Electrolyte swelling is depicted in Figure 4a. It is quite evident that the hydrogel discs exhibit lesser swelling in the CaCl_2_ solution compared to the NaCl solution at the same concentration. This might be attributed to the higher affinity of calcium ions toward their counter ions that are present in the polymeric chains.

### 3.5. pH-Responsive Swelling–Deswelling Studies

To scrutinize the stimuli sensitivity of the fabricated hydrogel discs, a pH-responsive swelling deswelling study was executed in a phosphate buffer with a pH of 7.4 and an acidic buffer with a pH of 1.2. The quince/mucin co-poly (methacrylate) hydrogel exhibits swelling in the phosphate buffer with a pH of 7.4, while deswelling is evident when the swollen disc was immersed in an acidic buffer with a pH of 1.2. The swelling–deswelling cycle was repeated thrice, as depicted in Figure 4b. The swelling response in the phosphate buffer with a pH of 7.4 is attributed to the repulsive forces between un-protonated carbonyl groups, while deswelling in an acidic medium is mainly due to protonation of carbonyl groups [14].

### 3.6. Sol–Gel Fraction

**Figure 5:**
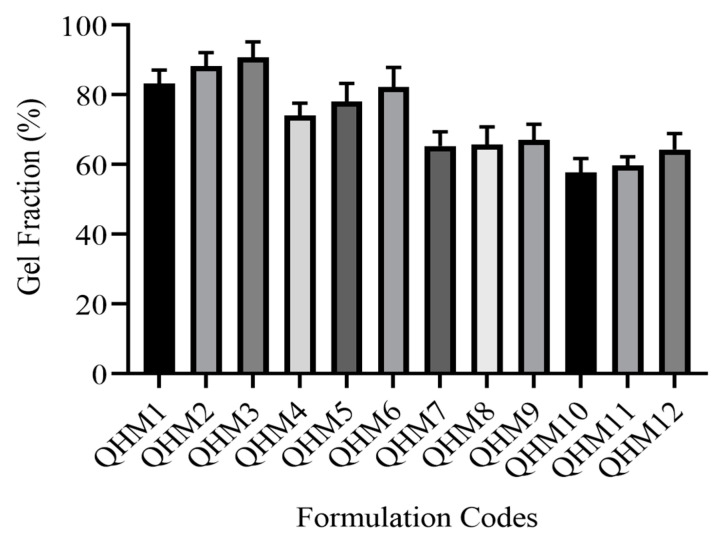
Gel fraction (%) of formulations QHM1-QHM12.

To establish the effect of quince, mucin, MAA and MBA on the sol and gel fraction (%) of the fabricated hydrogel, the discs with different formulations (QHM1–QHM12) were immersed in deionized water at 37 °C for 36 h. The rise in the gel fraction from 85 to 95% with increasing QH is evident in formulations QHM1–QHM3, as displayed in Figure 5. A similar tendency was witnessed when the amount of mucin was increased. In this case, the gel fraction increases from 75 to 85% in the formulations QHM4–QHM6. A subsequent rise in the gel fraction from 65 to 68% was witnessed in the formulations QHM7–QHM9 with increasing concentrations of MAA. This rise in the gel fraction with increasing concentrations of polymers and monomers could be attributed to an increase in carbonyl groups, leading to an increase in reactive sites on the polymers. The subsequent escalations in reactive sites lead to a stronger interaction between the polymers and the monomers, and thus the gel contents are augmented. Likewise, in formulations QHM10-QHM12 by increasing MBA contents gel fraction was pronounced from 57.66% to 64.33%. This was due to higher crosslinking density of the network due to incremental rise of MBA contents.

### 3.7. In Vitro Drug Release Studies

**Figure 6:**
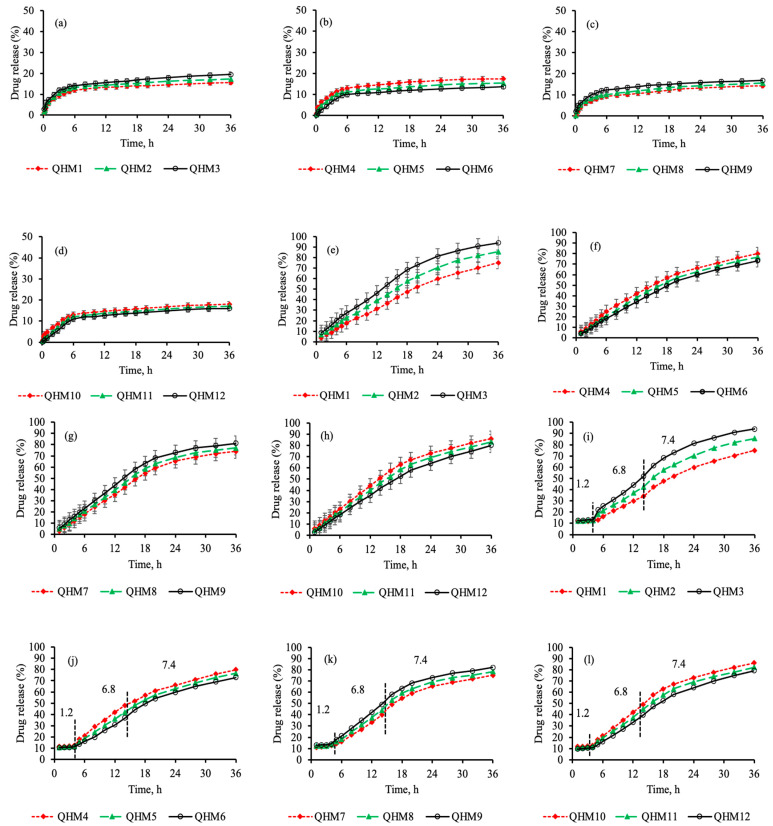
Drug release (%) of the formulations (QHM1-QHM12) in an acidic buffer with a pH of 1.2 (**a**–**d**), in a phosphate buffer with pH of 7.4 (**e**–**h**), and in a condition that mimics the pH of different segments of the intestine (**i**–**l**).

Drug release from the quince/mucin co-poly (methacrylate) hydrogel discs was ascertained in an acidic buffer with a pH of 1.2 and phosphate buffer with a pH of 6.8 or 7.4 for 36 h, as show in Figure 6a–d. A minor amount (15.23–21.77%) of the drug was released from all formulations (QHM1–QHM12) in the acidic buffer for 36 h; however, a sustained pattern of drug release was witnessed in the phosphate buffer with a pH of 6.8 or 7.4. The percentage of drug released for different formulations ranges from 71.04 to 86.99% and from 75.04 to 93.99% in the phosphate buffer with a pH of 6.8 and 7.4, respectively, as shown in Figure 6e–h. A similar pattern of drug release was observed when dissolution was performed in conditions that mimicked the pass-on time from various segments of the GIT [49,50]. 

The minor release of acyclovir sodium in an acidic medium is attributed to the negligible swelling of the hydrogel discs in acidic pH; as a result, the penetration of the dissolution medium into the polymeric matrices is hindered, resulting in lower drug release. In contrast, in the case of the buffer solutions with a pH of 6.8 or 7.4, drug release is increased due to higher swelling of the polymeric discs in the media. The pores in the hydrogel discs open up, allowing the penetration of the dissolution medium into the polymeric matrices; as a result, the drug release is geared up. 

The effect of quince, mucin, MAA and MBA on drug release was also scrutinized. The increase in the concentration of quince in the formulations QHM1–QHM3 results in an acceleration of drug release from 75.04 to 93.99% in these formulations, as shown in Figure 6i. This could be attributed to an increase in the number of carbonyl groups in the polymeric network, which results in enhanced swelling due to an increase in repulsive forces in the carbonyl groups and, ultimately, increases the drug release [41]. 

A similar effect on drug release was witnessed by increasing the concentrations of mucin (QHM4–QHM6) and MAA (QHM7–QHM9), as shown in Figure 6j,k. This increment in drug release is accredited to an increase in carboxylic and hydroxyl groups within the polymeric network, which intensifies the repulsive forces within the polymeric network and promotes acyclovir sodium release. Acyclovir sodium release declines from 85.99 to 80.11% with increasing concentration of the cross-linker in the formulations QHM10–QHM12, as shown in Figure 6l.

Different kinetic models were applied to the drug release data, and the regression coefficient (*R^2^*) was obtained using the DDSolver add-in program. The highest values were obtained for the Hixen–Crowell model, which governs the drug release from cylindrical systems (0.994–0.999). To realize the mechanism of drug release from different formulations, the Korsmeyer–Peppas equation was used. The value of the exponent “*n*” was determined. For all formulations, the value of “*n*” was calculated to be 0.689–0.867, which depicts anomalous or non-Fickian diffusion (Table 2). The results of the dissolution studies of the quince/mucin co-poly (methacrylate) hydrogel comply with the findings of earlier investigations, in which pH-responsive methacrylate hydrogels were incorporated to attain controlled release of loaded moieties for prolonged period of time [51,52,53].

### 3.8. Scanning Electron Microscopy

**Figure 7:**
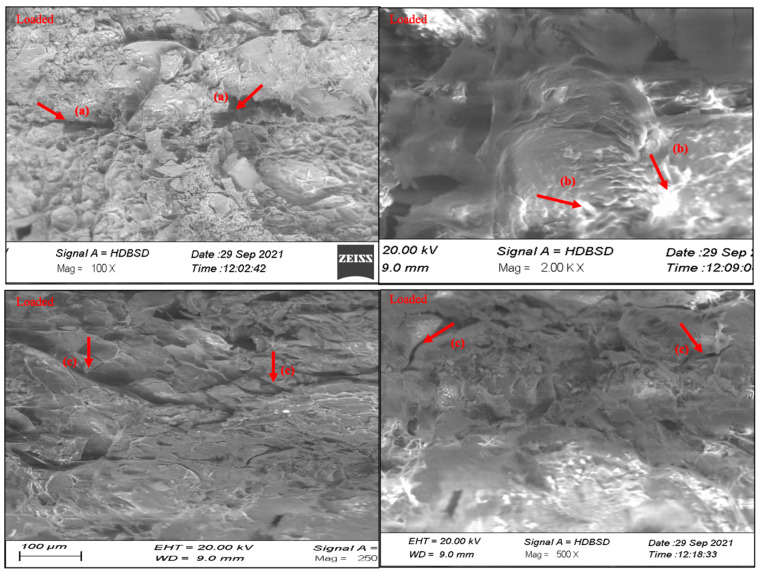

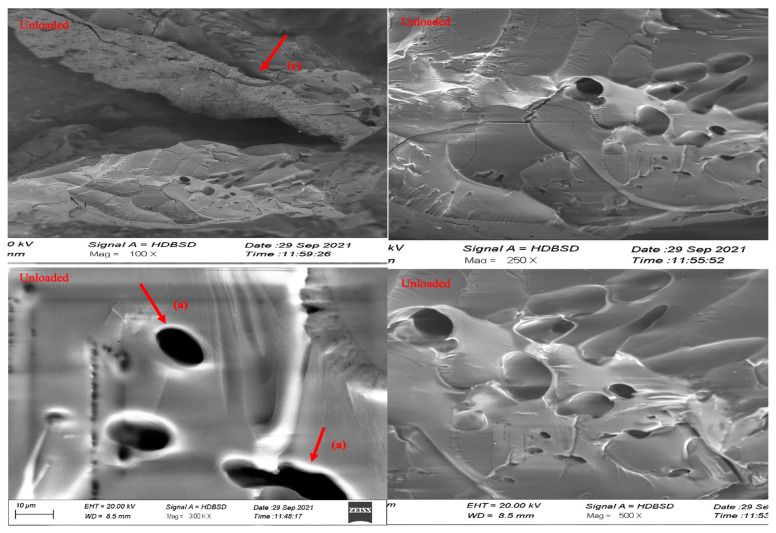
SEM images of unloaded and acyclovir sodium-loaded discs showing the presence of pores (a) Drug present on the surface (b) and cracks on the surface of the discs (c).

SEM was used to analyze the surface morphology of the optimized hydrogel formulations. The SEM images of the surface and the cross-sections of swollen freeze-dried loaded or unloaded hydrogel discs at different magnifications were recorded. Figure 7 depicts the surface morphology of an intact and cross-sectional hydrogel disc, which demonstrates that the co-polymeric hydrogel has a dense mass and a rough surface. This is due to the high cross-linking between MAA and MBA constituents [54]. The SEM images reveal the presence of capillary channels in the hydrogel discs, which accounts for the high swelling profile of the hydrogel discs [55]. The SEM images also clarify the drug loading into the polymeric matrices. 

### 3.9. Drug-Excipient Compatibility Studies

**Figure 8:**
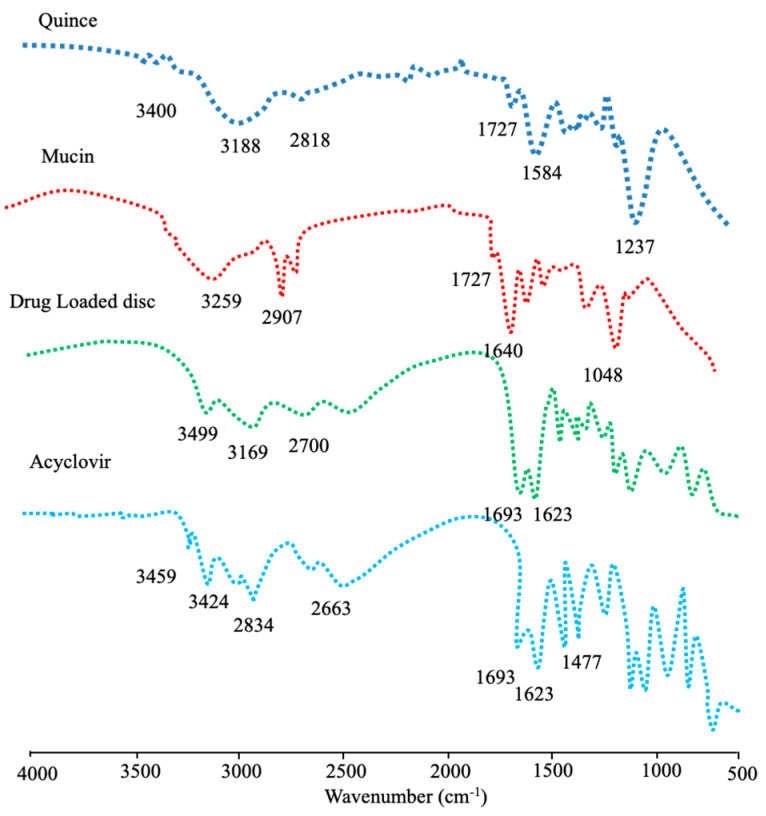
FTIR spectra of a quince, mucin, drug–loaded disc and acyclovir sodium.

The FTIR spectrum of a quince-, mucin-, drug-loaded disc and acyclovir sodium are shown in Figure 8. The FTIR spectrum of the QH as a biopolymer shows a peak appearing at 3400–3500 cm^−1^ that pertains to the OH groups, while the peaks at 3100–2700 cm^−1^ are due to the (-CH) aliphatic stretch. The intensive peak appearing at 1727.09 cm^−1^ indicates the presence of a carbonyl group in the polymeric chain of the QH, while the peaks at 1584.40 cm^−1^ show the presence of (C-O-C) groups, confirming the polysaccharides-based polymers.

The FTIR spectrum of mucin shows peaks at 3421.83–3259.24 cm^−1^ due to the N-H stretch, indicating the presence of an amide group, while the peaks appearing at 2900–2700 cm^−1^ pertain to CH- stretching. The major peak at 1727 cm^−1^ is due to the presence of the carbonyl group (C=O), while the peaks at 1640.48 cm^−1^ are due to N-H bending.

The FTIR spectrum of acyclovir sodium shows principal transmittance peaks at 3499 cm^−1^ due to the presence of hydroxyl (-OH) group and peaks at 3458.03–3100 cm^−1^ due to the (-NH) stretch, while the peaks at 2873 cm^−1^ indicate CH stretch. A peak appearing at 1693 cm^−1^ pertains to the presence of a carbonyl (C=O) group, the peak at 1477 cm^−1^ represents (NH) bending in the secondary amines, and the peak at 1175 cm^−1^ represents the presence of (C–N) stretch.

The characteristic peaks of acyclovir sodium were found intact in the drug-loaded hydrogel discs, establishing the compatibility of the drug with polymers. Minor shifting in the peaks of the polymers and the appearance of a few newer peaks might be attributed to the free-radical polymerization.

### 3.10. Powder X-ray Diffraction Analysis

**Figure 9:**
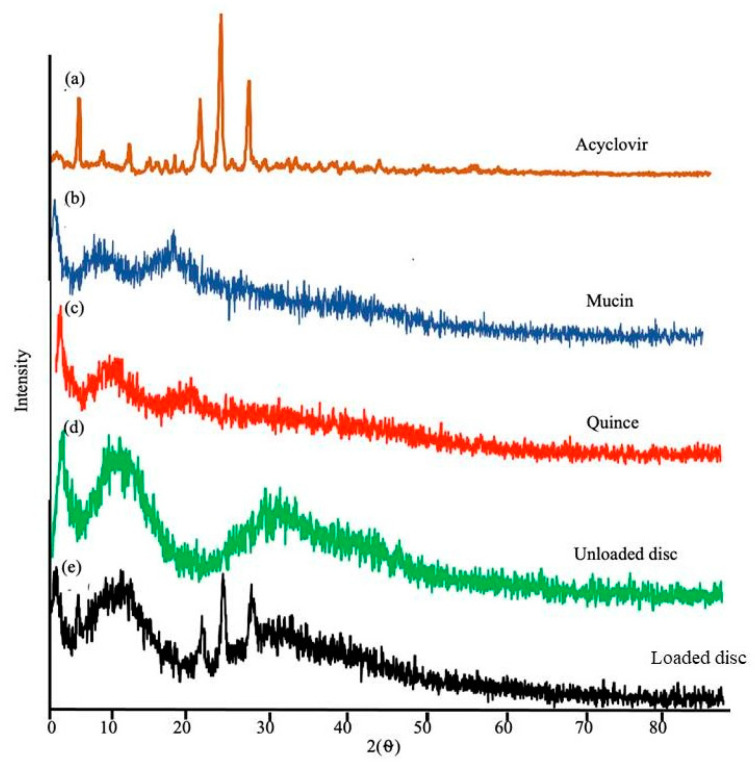
Powdered X-ray diffractograms of acyclovir sodium (**a**), mucin (**b**), quince (**c**), unloaded disc (**d**) and loaded disc (**e**).

Powder X-ray diffraction (PXRD) analysis was carried out to spot the amorphous or crystal nature of the pure drug, the polymers, the unloaded hydrogel discs and the drug-loaded hydrogel discs. The PXRD pattern of acyclovir sodium shows intensive peaks at 20° and 30°, accounting for the crystalline nature of acyclovir sodium [50,51]. The diffractogram of the individual polymers and the hydrogel discs does not have any sharp peaks, which reflect their amorphous nature, as shown in Figure 9. The PXRD for the acyclovir sodium-loaded hydrogel discs exhibits few sharp peaks, indicating the transition of acyclovir sodium from a crystalline to a semi-crystalline form after loading into the hydrogel matrices. This transition from the crystalline to semi-crystalline form of acyclovir sodium is beneficial in improving its water solubility.

### 3.11. Thermal Analysis 

**Figure 10:**
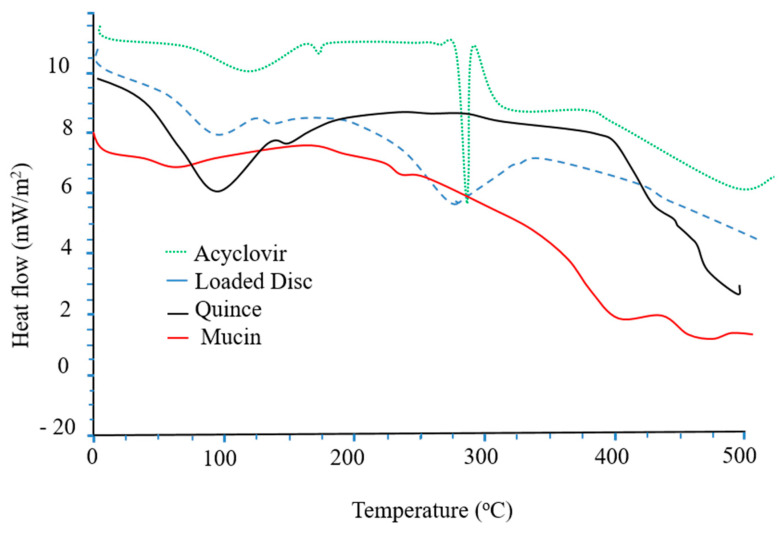
DSC thermograms of acyclovir sodium, loaded disc, quince and mucin.

The thermal stability of the hydrogel discs, as well as the polymers and acyclovir sodium before and after loading in the hydrogel discs, was tested using differential scanning calorimetry (DSC). The DSC of acyclovir sodium shows two peaks; an initial endothermic peak at 120 °C due to the loss of moisture from the drug, while the second endothermic phase transition at 260 °C pertains to the melting point of acyclovir sodium [47]. The DSC of the QH exhibits an initial endothermic peak at 100–150 °C (Figure 10). Two phases of thermal degradation are evident in the DSC of the QH. In the first phase of degradation, an endothermic peak appears at 200–400 °C, while the second stage is witnessed at 400–600 °C. This degradation is due to the decomposition of residual cellulose [48]. In the DSC of mucin, endothermic peaks appear at 100 °C and at 250–400 °C. These peaks pertain to moisture loss and breakage of glycosidic linkage of the polymeric chain, respectively. The DSC thermogram of the drug-loaded formulation indicates a shifting of the endothermic peaks toward a higher temperature, which reflects the improved thermal stability of the formulation compared to the individual components. 

### 3.12. In Vivo X-ray Study

**Figure 11:**
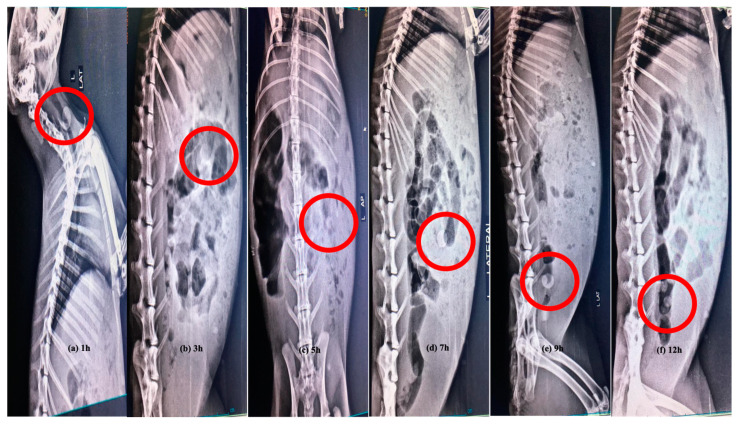
In vivo real-time X-ray images of a hydrogel disc in different segments of the GI tract at different time intervals (**a**) 1 h, (**b**) 3 h, (**c**) 5 h, (**d**) 7 h, (**e**) 9 h and (**f**) 12 h. Red circles specify the position of hydrogel disc.

After the oral administration of the quince/mucin co-poly (methacrylate) hydrogel discs to the white albino rabbits, radiographical images were captured to investigate the physical condition of the hydrogel disc during transit from the GIT. During the 12 h study period, the passage of the hydrogel disc through different segments of GIT, i.e., stomach, small intestine and colon, is evident. During the first hour, sharp and bright images of the disc show no sign of disintegration, as well as no visible sign of swelling, indicating that the disc is intact in the stomach of the rabbit. The images captured after 4–5 h depict an increase in the size of the disc, accounting for the swelling of the disc within the upper part of the small intestine. After 9 h, the radiographical image clearly indicates the presence of the disc in the large intestine, where the size of the disc increases due to swelling. After 12 h, the presence of the disc is evident in the colon of the rabbit (Figure 11), where it is quite evident that the disc is still intact and still possesses the same integrity to provide drug release for a prolonged period of time.

### 3.13. Acute Oral Toxicity Studies

To appraise the safety profile of the newly developed hydrogel carrier system, acute toxicity studies were performed on white albino rabbits. During these studies (14 days), the animals were kept in a controlled environment, and they were strictly monitored for various parameters, such as body weight, food and water intake, behavioral changes and other illnesses, such as dermal and ocular allergies.

**Figure 12:**
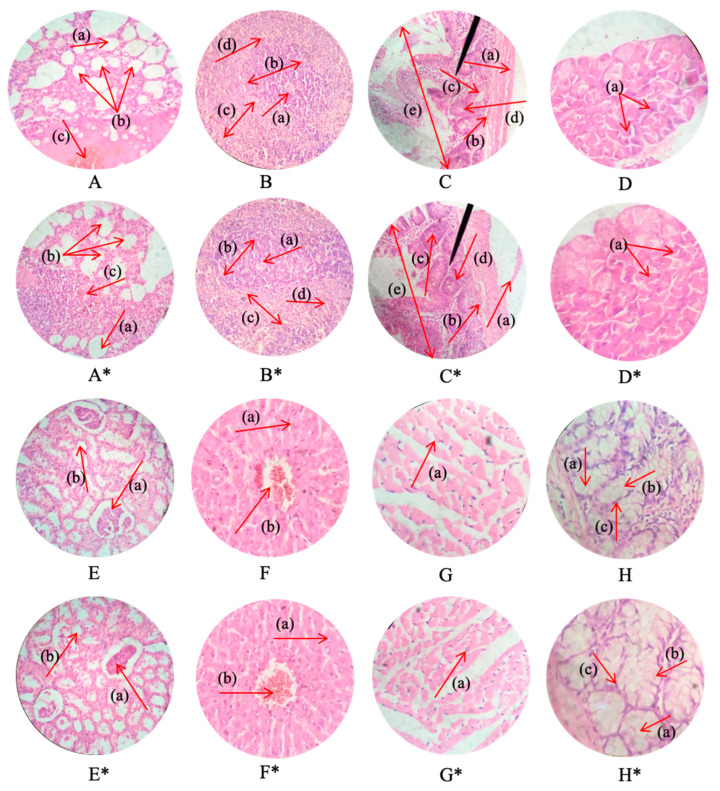
Histopathology of lungs (**A**,A*): alveolus (a), alveoli (b), and blood vessels (c). Spleen (**B**,B*): central arteriole (a), white pulp (b), red pulp (c), and trabecular (d). Small intestine (**C**,C*): lamina propria (a), muscularis mucosae (b), acinous lumen (c), columnar epithelial cell with basal nuclei (d), and small intestinal villi (e). Pancreas (**D**,D*): pancreatic acini (a). Kidney (**E**,E*): glomerulus (a) and renal tubules (b). Liver (**F**,F*): plates of hepatocytes (a) and blood vessels (b). Heart (**G**,G*): cardiac muscle fibers (a). Colon (**H**,H*): lumen of crypt (a), colon crypt (b), and lamina propria (c). A*,B*,C*,D*, E*,F*,G* and H* represent the histopathological images of the lungs, spleen, small intestine, pancreas, kidney, liver, heart, and colon of the treated animals. All images were captured at 40x magnification.

No sign of illness in any animal of both the control and treated groups was observed after the administration of the hydrogel disc. No significant differences were noticed in terms of body weight and food and water consumption between the treated and control groups. Moreover, the behavioral pattern of both the treated and control animals were observed to be normal, and no signs of dermal as well as ocular allergies were noticed. No incidence of mortality was documented in all three groups. The results of the toxicity studies are presented in Table 3 and Table 4. The findings of the blood sample analyses of both control and treated animals are comparable and within normal ranges, establishing the non-toxic nature and biocompatibility of the newly fabricated hydrogel. The results of hematological and biochemical parameters are presented in Table 5 and Table 6. To appraise the effect of the fabricated hydrogel discs on the cellular architecture of vital organs, histopathological studies were carried out. The results of the histopathological studies reveal the normal cellular architecture of vital organs, such as the liver, heart, kidney, spleen, intestine, lungs and brain (Figure 12). No signs of inflammation were observed in the cellular architecture of vital organs. The cardiac myocytes of the heart segment were well organized, retaining their structural geometry and exhibiting no tissue damage. The liver cells did not present any sort of degradation. The hepatic cord and lobules were properly arranged. The lung tissues did not show any sort of hemorrhagic site, the alveoli were normal with thick wall, and there was no fluid accumulation within the lumen. The spleen tissues did not exhibit any sort of splenomegaly, and the red and white zones were clearly distinct. No sign of hyperplasia and ulceration was observed in the intestinal segment. In the kidney tissue, there was no fibrosis, and the Bowman’s capsule and other components were distinguishable. These findings reveal the non-toxic nature of the developed hydrogel system.

## 4. Conclusions

The Quince/mucin co-poly (methacrylate) hydrogel was successfully fabricated using the free-radical polymerization technique. The graft copolymer manifested stimuli-responsiveness effect. The hydrogel network provided acyclovir sodium release at a controlled rate, releasing 75.04 to 93.99% during 36 h in a buffer solution with a pH of 7.4. Moreover, a minor amount of the drug was released in an acidic buffer with a pH of 1.2. The FTIR analysis confirmed the compatibility among the different formulation components. The thermal analysis proved the thermal stability of the carrier system. The SEM analysis presented rough surfaces embedded with pores and cracks. The PXRD analysis depicted the amorphous nature of the prepared hydrogel network. The acute toxicity results proved that the fabricated hydrogel network was biocompatible, showing non-toxic behavior. No changes in hematological and biochemical parameters were noticed. The histopathology of the cellular network displayed no sign of abnormality. Considering the results, it could be inferred that the quince/mucin co-poly (methacrylate) hydrogel could be used as a potential carrier for controlled drug delivery, which would be helpful in reducing unwanted GIT effects and improving patient compliance as well as therapeutic output. 

## Figures and Tables

**Table 1:** Compositions of different formulations of quince/mucin co-poly (methacrylate) hydrogels.

Code	Quince (mg/5 mL)	Mucin (mg/5 mL)	MAA (g/5 mL)	APS (g/5 mL)	MBA (g/5 mL)
QHM1	50	50	3	0.1	0.2
QHM2	100	50	3	0.1	0.2
QHM3	150	50	3	0.1	0.2
QHM4	100	75	3	0.1	0.2
QHM5	100	100	3	0.1	0.2
QHM6	100	150	3	0.1	0.2
QHM7	100	50	4	0.1	0.2
QHM8	100	50	5	0.1	0.2
QHM9	100	50	6	0.1	0.2
QHM10	100	50	4	0.1	0.3
QHM11	100	50	4	0.1	0.5
QHM12	100	50	4	0.1	0.7

MAA: methacrylic acid; APS: ammonium persulfate; MBA: *N,N′*-methylene-bis-acrylamide.

**Table 2:** Values of the regression coefficient (*R^2^*) for different kinetic models.

Kinetic Models	Regression Coefficient	QHM1	QHM2	QHM3	QHM4	QHM5	QHM6	QHM7	QHM9	QHM10	QHM12
Zero-order	*R* ^2^	0.986	0.985	0.970	0.928	0.986	0.982	0.990	0.982	0.990	0.979
First order	*R* ^2^	0.986	0.980	0.982	0.997	0.994	0.995	0.993	0.990	0.992	0.991
Higuchi model	*R* ^2^	0.859	0.905	0.933	0.944	0.912	0.901	0.923	0.892	0.922	0.893
Korsmeyer–Peppas	R^2^	0.988	0.990	0.985	0.996	0.983	0.987	0.980	0.988	0.979	0.987
	*n*	0.867	0.766	0.69	0.689	0.734	0.769	0.701	0.793	0.701	0.788
Hixen–Crowell	*R* ^2^	0.994	0.994	0.995	0.999	0.994	0.996	0.995	0.997	0.995	0.997

R^2^: regression coefficient; *n*: Korsmeyer–Peppas exponent.

**Table 3:** Assessment of body weight of the control and treated groups of rabbits.

Animal Groups	Group A (Control)	Group B (Treated, 2 g/kg)	Group C (Treated, 3 g/kg)
	Mean ± SEM	Mean ± SEM	Mean ± SEM
Body weight (g)			
Pre-treatment	1444 ± 25.4	1376 ± 31.4	1351 ± 31.8
Day 1	1419 ± 33.8	1377 ± 30.5	1352 ± 28.3
Day2	1421 ± 31.5	1345 ± 28.5	1368 ± 25.7
Day3	1409 ± 26.8	1352 ± 25.1	1337 ± 28.3
Day5	1401 ± 29.2	1352 ± 31.2	1352 ± 21.7
Day 7	1425 ± 28.2	1368 ± 28.9	1352 ± 22.1
Day9	1430 ± 26.5	1366 ± 28.6	1363 ± 23.5
Day 11	1432 ± 29.4	1390 ± 31.6	1367 ± 28.3
Day 14	1439 ± 30.5	1388 ± 25.7	1371 ± 30.2

**Table 4:** Food and water intake of the control and treated groups of rabbits.

Parameters	Group A	Group B	Group C
Water intake (mL)	Mean ± SEM	Mean ± SEM	Mean ± SEM
Pre-treatment	19.1 ± 1.12	18.8 ± 2.11	19.1 ± 2.05
Day 1	19.3 ± 1.41	16.2 ± 1.22	16.7 ± 2.16
Day 2	19.5 ± 1.19	17.7 ± 1.92	17.9 ± 2.13
Day 3	19.3 ± 1.31	16.2 ± 1.64	16.2 ± 2.26
Day 4	19.7 ± 1.21	17.0 ± 2.12	18.8 ± 2.66
Day 14 Food Intake (g)	19.6 ± 1.41	17.2 ± 2.32	17.5 ± 2.82
Pre-treatment	16.8 ± 1.41	17.2 ± 1.02	16.1 ± 2.1
Day 1	16.9 ± 1.23	16.0 ± 0.91	16.8 ± 1.6
Day 2	17.0 ± 1.36	16.2 ± 1.04	18.6 ± 1.7
Day 3	17.2 ± 1.76	16.5 ± 1.40	17.2 ± 1.8
Day 4	18.1 ± 1.76	16.0 ± 1.26	16.8 ± 1.2
Day 14	19.0 ± 2.05	19.8 ± 1.36	17.5 ± 2.1

**Table 5:** Hematological parameters of the control and treated groups of rabbits.

Hematological Parameters	Normal Ranges	Group A (control)	Group B (2 g/kg)	Group C (3 g/kg)
CBC *				
TLC *	8.1–21.5 × 10^3^/µL	14.25 ± 0.06	11.21 ± 0.15	10.15 ± 0.13
RBC *	3.8–7.9 × 10^6^/µL	5.28 ± 0.04	4.16 ± 0.03	4.78 ± 0.08
Hb *	9.4–17.4 g/dL	12.61 ± 0.04	11.26 ± 0.05	14.29 ± 0.10
HCT *	35–40%	36.41 ± 1.12	37.57 ± 0.52	38.17 ± 0.08
MCV *	50–75 fL	56.31 ± 1.24	64.83 ± 1.01	56.03 ± 1.56
MCH *	18–24 pg	20.11 ± 0.75	18.87 ± 1.01	20.08 ± 0.50
MCHC *	27–34 g/dL	30.05 ± 2.05	28.13 ± 0.21	29.14 ± 0.45
Platelet Count	250–650 × 10^3^/µL	345.47 ± 3.60	284.63 ± 2.94	399.22 ± 3.46
Neutrophils	34–70%	43.49 ± 1.51	39.57 ± 1.76	47.12 ± 0.40
Lymphocytes	30–70%	50.67 ± 1.20	37.71 ± 1.41	43.03 ± 0.71
Monocytes	0–3%	1.28 ± 0.11	1.05 ± 0.03	1.66 ± 0.11
Eosinophils	0–1%	0.34 ± 0.01	0.70 ± 0.01	0.55 ± 0.01

* CBC: complete blood count; TLC: total leukocyte count; RBC: red blood cells; Hb: hemoglobin; HCT: hematocrit test; MCV: mean corpuscular volume; MCH: mean corpuscular hemoglobin; MCHC: mean corpuscular hemoglobin concentration.

**Table 6:** Biochemical parameters of the control and treated groups of rabbits.

Blood Parameters	Normal Ranges	Group A	Group B	Group C
ALT (U/I) *	Less than 34	24.25 ± 0.06	21.21 ± 0.15	30.15 ± 0.13
AST (U/I) *	Up to 31	25.28 ± 0.04	24.16 ± 0.03	24.78 ± 0.08
Alkaline Phosphate (U/I)	65–304	32.61 ± 0.04	31.26 ± 0.05	34.29 ± 0.10
Albumin (g/dL)	3.5–5.0	32.41 ± 1.12	31.57 ± 0.52	28.17 ± 0.08
Globulin (g/dL)	2.5–3.5	26.31 ± 1.24	34.83 ± 1.01	26.03 ± 1.56
Triglycerides (µmol/L)	Desirable: <200 Borderline: 200–400 Elevated: >400	30.11 ± 0.75	28.87 ± 1.01	30.08 ± 0.50
HDL (mg/dL) *	Low: <50 High: >60	30.05 ± 2.05	28.13 ± 0.21	29.14 ± 0.45
VLDL (mg/dL) *	<30	32.47 ± 3.60	28.63 ± 2.94	27.22 ± 3.46
Urea (mg/dL)	28–45	28	30	28
Creatinine (mg/dL)	1.47–3.9	0.9	1.1	1.2

* ALT: alanine transaminase; AST: aspartate aminotransferase; HDL: high-density lipoproteins; VLDL: very-low-density lipoproteins.

## Data Availability

Research data can be provided by the corresponding authors upon reasonable request.
